# Assessing patient information needs for new antidiabetic medications to inform shared decision‐making: A best–worst scaling experiment in China

**DOI:** 10.1111/hex.14059

**Published:** 2024-04-30

**Authors:** Tongling Xie, Jingyi Meng, Zhe Feng, Yue Gao, Tian Chen, Yalan Chen, Jinsong Geng

**Affiliations:** ^1^ Center for Evidence‐Based Medicine Nantong University Medical School Nantong China; ^2^ Information Center The People's Hospital of Rugao Nantong China; ^3^ Hepatobiliary Center The First Affiliated Hospital of Nanjing Medical University Nanjing China; ^4^ Department of Rehabilitation Medicine and Clinical Medicine Medical Institute of Taizhou Polytechnic College Taizhou China

**Keywords:** best–worst scaling, diabetes, information needs, shared decision‐making

## Abstract

**Background:**

Shared decision‐making (SDM) is a patient‐centred approach to improve the quality of care. An essential requirement for the SDM process is to be fully aware of patient information needs.

**Objectives:**

Our study aimed to assess patient information needs for new antidiabetic medications using the best–worst scaling (BWS) experiment.

**Methods:**

BWS tasks were developed according to a literature review and the focus group discussion. We used a balanced incomplete block design and blocking techniques to generate choice sets. The final BWS contains 11 attributes, with 6‐choice scenarios in each block. The one‐to‐one, face‐to‐face BWS survey was conducted among type 2 diabetic patients in Jiangsu Province. Results were analyzed using count‐based analysis and modelling approaches. We also conducted a subgroup analysis to observe preference heterogeneity.

**Results:**

Data from 539 patients were available for analysis. The most desired information domain was the comparative effectiveness of new antidiabetic medications. It consists of the incidence of macrovascular complications, the length of extended life years, changes in health‐related quality of life, the incidence of microvascular complications, and the control of glycated haemoglobin. Of all the attributes, the incidence of macrovascular complications was the primary concern. Patients' glycemic control and whether they had diabetes complications exerted a significant influence on their information needs.

**Conclusions:**

Information on health benefits is of critical significance for diabetic patients. Patients have different information needs as their disease progresses. Personalized patient decision aids that integrate patient information needs and provide evidence of new antidiabetic medications are worthy of being established.

**Patient or Public Contribution:**

Before data collection, a pilot survey was carried out among diabetic patients to provide feedback on the acceptability and intelligibility of the attributes.

## 1 INTRODUCTION

Diabetes is a chronic metabolic disorder characterized by persistent hyperglycaemia. Over time, diabetes can cause serious damage to the heart, kidneys, eyes, and nerves. Diabetes has been a major public health problem worldwide. The global diabetes prevalence is estimated to rise to 10.2% (578 million) by 2030 and 10.9% (700 million) by 2045.^1^ With over 114 million adults living with diabetes (predominately type 2 diabetes), China has the unwanted claim of being the epicentre of the diabetes crisis. Diabetes imposes a heavy social burden not only on health and well‐being but also on productivity. In 2017, an estimated 7.1% (56.4 million) of the working‐age population in China had diabetes, resulting in an average of 1.3 productivity‐adjusted life years lost per person.^2^

With diabetes continuing to be a growing problem across the world, there is an ongoing need for new medications to help control blood glucose levels. In recent years, therapeutic discoveries of new antidiabetic medications have expanded the choices for treatment strategies. However, a clinical decision regarding the best available therapeutics could be complex due to the heterogeneity of individual needs and preferences among diabetic patients. Shared decision‐making (SDM) has been advocated as an optimal approach to achieving patient‐centred care.^3^, ^4^, ^5^ It is a collaborative process where clinicians and patients share the best available evidence when making decisions and where patients are supported to consider therapeutic options.^6^

Effectively engaging patients in their healthcare decisions is essential to improve patient satisfaction with healthcare services and new health technologies.^7^, ^8^, ^9^, ^10^ Therefore, SDM is appropriate for diabetes care and has the potential to enhance the quality of care.^11^ To practice SDM, patients need information about their medical conditions and the value of antidiabetic medications. Although several studies have been conducted on the information needs of diabetic patients, they focused on the topics of health education, lifestyle adjustment and healthcare management.^12^, ^13^, ^14^ A systematic review found that information needs of diabetic patients in the published literature included treatment process, course of disease, abnormalities in glucose metabolism, and diabetes throughout the life cycle.^15^ Existing studies rarely pay attention to the information needs of patients for new antidiabetic medications. Furthermore, clinicians are usually unaware of what information the patients want and often ignore patient values, which hinders the process of SDM.^16^, ^17^

The traditional approach to measure information needs was through the application of rating scales. However, the use of rating scales does not always result in reliable findings due to response style biases such as social desirability bias, extreme response bias, and acquiescence bias.^18^, ^19^ Best–worst scaling (BWS), also known as maximum‐difference scaling, consists of choice tasks in which a respondent is asked to indicate the best and the worst options. It is an attractive method used in preference‐based studies on a wide range of healthcare topics.^20^, ^21^ A comparison of the BWS and rating scales found that the BWS approach helps to validate priorities from a respondent perspective by achieving better discrimination among attributes.^22^ BWS might outperform rating scales in terms of both the ability to predict preferences and test‐retest reliability.^23^, ^24^

To address the evidence gap, we aimed to investigate the patient information needs of new antidiabetic medications using the BWS experiment. Our findings would inform clinicians to provide patients with the desired information, thus promoting the implementation of SDM. The results would also contribute to the development of patient decision aids (PDAs) that are tailored to patient information needs.

## 2 METHODS

### 2.1 Experimental design

There are three types of BWS experiments: object case (Case 1), profile case (Case 2) and multiprofile case (Case 3). Case 1 is a classic case of BWS. In Case 1 BWS, individuals are asked to choose the best and worst from a set of objects. In Case 2 BWS, the level of each attribute is shown, and the choice set has the structure of a single profile. Case 3 BWS constructs various combinations of attribute levels (profiles) and then asks respondents to select the best and worst profiles in each choice set. The attributes of health technologies and healthcare services can be complicated. Case 1 BWS is attractive in healthcare because it does not assess trade‐offs among different preference levels. Our study used Case 1 BWS, which is ideal for incorporating a relatively large number of factors and is considered to be less cognitively burdensome, particularly among vulnerable patient groups.^25^, ^26^ It is an effective alternative to traditionally used ranking or rating tasks.^27^, ^28^

### 2.2 Identification of attributes

In this study, we defined new antidiabetic medications as new classes of antidiabetic agents, for example, glucagon‐like peptide‐1 receptor agonists, sodium‐glucose co‐transporter 2 inhibitors and dipeptidyl peptidase 4 inhibitors. We did not restrict new antidiabetic medications to specific drugs due to emerging therapeutic drug targets and treatment options in the near future. We took a three‐step approach to define the attributes in our BWS survey. First, a literature review was conducted on 20 December 2021, to identify value assessment frameworks for new health technologies, including antidiabetic therapies. Details of the included frameworks are shown in Supporting Information S1: Appendix 1. We also searched for preference‐based studies to identify potential attributes that could assess the value of antidiabetic therapies. Attributes in the included studies are shown in Supporting Information S1: Appendix 2.

Second, due to the vast universe of attributes, we conducted a focus group discussion with six clinicians to further determine the attributes. The question guide for the focus group discussion is shown in Supporting Information S1: Appendix 3. According to our literature review, the patient‐reported outcome has been regarded as an essential domain in the value‐assessment frameworks for health technologies. Health‐related quality of life (HRQoL) is a multidimensional concept widely used to examine the impact of health status on quality of life. Diabetes and its complications adversely influence patients’ HRQoL.^29^, ^30^ Therefore, we also used HRQoL as a patient‐reported outcome measure.

Finally, a pilot survey with 20 diabetic patients was conducted to provide feedback on the acceptability and intelligibility of the descriptions of attributes. Responses from patients led to a more apprehensible statement of survey questions, including the explanations of choice scenarios in survey manuals. Attributes in our BWS are listed in Table 1.

**Table 1:** Domains and attributes in the best–worst scaling experiment.

Domains of information needs	Attributes to reflect information needs
Information on comparative effectiveness	Control of HbA_1c_
	Changes in HRQoL
	Incidence of macrovascular complications
	Incidence of microvascular complications
	Length of extended life years
Information on comparative safety	Incidence of severe hypoglycaemia events
	Incidence of gastrointestinal side events
	Weight change
Information on convenience	Pill burden
	Dosing frequency
Information on affordability	Out‐of‐pocket costs

Abbreviations: HbA_1c_, haemoglobin A_1c_; HRQoL, health‐related quality of life.

### 2.3 Experimental design and development of the questionnaire

A balanced incomplete block design, the most commonly used design for Case 1 BWS, was applied to construct choice sets. Choice sets were established using R 4.2.0 (R Foundation for Statistical Computing). We employed the blocking technique to divide choice sets into four blocks, with each block having six scenarios and each scenario containing four options. Respondents were asked to select the most needed (most important) and the least needed (least important) information about new antidiabetic medications when making clinical decisions. The questionnaire for the BWS experiment is demonstrated in Supporting Information S1: Appendix 4.

The questionnaire contained two parts. The first part consisted of patients' sociodemographic characteristics, medical history, clinical features and attitudes towards SDM. Patients' medical history and clinical features were fulfilled by interviewers utilizing information from electronic medical records. Patients should respond to three closed‐ended questions, including ‘I am fully aware of my antidiabetic medications’, ‘I am willing to actively engage in the SDM process for antidiabetic medications’ and ‘I am willing to know the value of new antidiabetic medications with the help of PDAs, which provide scientific evidence’. A 5‐point Likert scale, ranging from 1 (strongly disagree) to 5 (strongly agree), was used as the evaluation method. The second part was the BWS tasks and patients' confidence when making their choices. The score ranged from zero (no confidence at all) to 10 (very confident). We excluded BWS questionnaires with an average score of less than 8 to ensure the validity of the data.

### 2.4 Sample size

Currently, there is no formal guidance on the optimal sample size for BWS.^21^, ^28^ The sample size is usually determined according to previous studies, rules of thumb and budget constraints.^31^, ^32^ Lancsar and Louviere proposed that more than 20 respondents per choice set could estimate reliable models.^33^ A recent systematic review showed the median sample size of BWS in healthcare was 220.^20^ Consequently, we had a minimum sample size of 500 to provide reasonable precision in our estimates.

### 2.5 Survey implementation and data collection

The BWS experiment was carried out from 1 January to 30 April 2022. There were five sampling hospitals in five cities in Jiangsu Province. The sample size was evenly distributed among the sampling hospitals. We enroled patients aged 18 years or older with a definite diagnosis of type 2 diabetes for at least 1 year and who took medications regularly. We excluded patients who had been diagnosed with gestational diabetes.

A total of 13 interviewers were recruited for our survey, including 7 postgraduate medical students and 6 physicians. To ensure the validity of the survey, our BWS questionnaires were administered through one‐to‐one, face‐to‐face interviews. For quality assurance, we compiled survey manuals and trained interviewers either face‐to‐face or online. The interviewers were asked to check the completeness of each questionnaire immediately after it was completed. For patients who had blurred vision or were illiterate, interviewers explained the questionnaire item by item until patients could clearly understand. The duration of the survey for each patient ranged from 15 to 30 min. Patients were told that participation in the survey was voluntary, and their written and informed consent was obtained before the survey. We gave each patient a wrapped cotton towel worth CNY10 as a gift.

### 2.6 Statistical analysis

First, a count‐based analysis was conducted to examine the frequency of choosing each attribute. The best–worst scaling score (B‐W score) was calculated as the number of times the attribute was selected as the most important minus the number of times that attribute was selected as the least important.^28^ Positive values of the B‐W score show that the attribute has been chosen more frequently as the ‘best’ (most preferred) than as the ‘worst’ (least preferred) option, and negative values indicate the opposite.

We calculated the standardized B‐W score to investigate the relative importance of each attribute using the following equation:

$$\mathrm{Standard}\;\mathrm{score}=\frac{{\mathrm{Count}}_{\mathrm{best}}{-\;\mathrm{Count}}_{\mathrm{worst}}}{r\times n},$$

where ${\mathrm{Count}}_{\mathrm{best}}$ equals the total number of times each attribute has been chosen as the ‘best’; ${\mathrm{Count}}_{\mathrm{worst}}$ is the total number of times each attribute has been selected as the ‘worst’; *r* represents the times each attribute appears; *n* is the number of observations.^34^ We also calculated the mean B‐W score using the total B‐W score as divided by the number of participants who responded to the attribute.

Second, we performed the sensitivity analysis by surface under the cumulative ranking (SUCRA) score to test the robustness of count‐based analysis. SUCRA has a natural scale from zero to one and can therefore be readily used as a weight.^35^ If an attribute is always ranked as the ‘worst’ option, it will receive a SUCRA score of zero. However, if it is always ranked as the ‘best’ option, it will receive a score of 1. The cumulative ranking curve of each outcome reveals the probability that an outcome has a certain rank.

Third, we did both the conditional logit regression and the multinomial logit regression to perform the individual‐level analysis. Conditional logit models the choice as a function of the choice characteristics, whereas multinomial logit models the choice as a function of the respondent's characteristics.^36^ The conditional logit regression uses a sequential best–worst assumption. Respondents are assumed to first select the most concerning option, followed by the least concerning option, in each choice set.^37^ Therefore, the main analysis was conditional logit regression. Multinomial logit regression was used as a sensitivity analysis to test the robustness of BWS estimates.

Finally, we conducted subgroup analysis by age, glycemic control, and whether patients had diabetes complications to observe preference heterogeneity in information needs. Data were analyzed using STATA 16.0 MP (StataCorp LLC).

## 3 RESULTS

### 3.1 Patients' characteristics and attitudes towards SDM

A total of 556 patients consented to participate in the BWS survey. Seventeen patients were excluded from the analysis due to noncompliance with the inclusion criteria, incomplete data, or a lack of confidence in choosing choice sets. As a result, data from 539 patients was available for analysis.

The sample consisted of more males than females (58.44% vs. 41.56%) (Table 2). The average age was 58.55 years old, ranging from 28 to 93 years old. A total of 336 patients (62.34%) had diabetes complications. Only 37.29% of patients had good glycemic control. Few patients (30.24%) were fully aware of their antidiabetic medications (2.52 ± 1.18). However, the vast majority of patients (79.04%) wanted to actively take part in the SDM process (4.00 ± 0.76). Most patients (74.40%) were willing to use PDAs to obtain evidence on the value of new antidiabetic medications (3.98 ± 0.89).

**Table 2:** Characteristics of diabetic patients (*N* = 539).

Variables	*N* (%)
Gender	
Male	315 (58.44)
Female	224 (41.56)
Age	
<65	345 (64.01)
65–74	117 (21.71)
≥75	77 (14.28)
Education	
Unschooled	53 (9.83)
Elementary school	125 (23.19)
Junior high school/high school	269 (49.91)
Junior college or above	92 (17.07)
Occupation	
Farmer	111 (20.59)
Urban employee	164 (30.43)
Retiree	158 (29.31)
Unemployed or freelancers	106 (19.67)
Monthly household income (Chinese Yuan [CNY])	
≤2000^a^	53 (9.83)
2001–4000	87 (16.14)
4001–6000	139 (25.79)
6001–8000	91 (16.88)
8001–10,000	93 (17.26)
>10,000	76 (14.10)
Glycemic control	
Good	201 (37.29)
Fair	234 (43.41)
Poor	104 (19.30)
Diabetes complications	
With	336 (62.34)
Without	203 (37.66)

^a^ The average exchange rate between US dollars and the CNY from July 2021 to June 2022 was 1:6.46. CNY 2000 was approximately US$309.60.

### 3.2 BWS estimates by count‐based analysis

**Figure 1:**
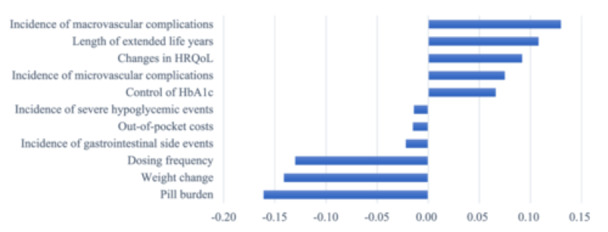
Standardized best‐minus‐worst scores for the attributes. HbA_1c_, haemoglobin A_1c_; HRQoL, health‐related quality of life.

**Figure 2:**
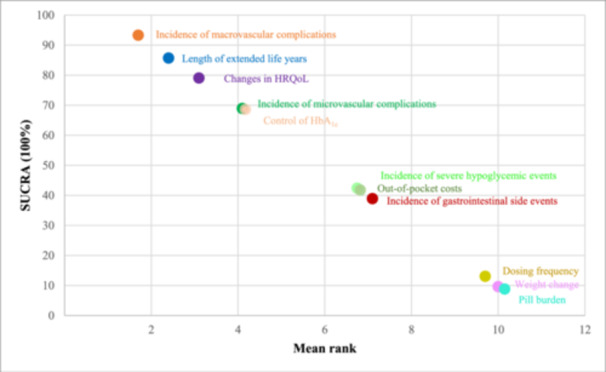
SUCRA plot and the mean rank of each attribute. HbA_1c_, haemoglobin A_1c_; HRQoL, health‐related quality of life; SUCRA, surface under the cumulative ranking.

The relative priority assigned to each attribute is represented by the standardized scores, as shown in Figure 1. The incidence of macrovascular complications, length of extended life years, changes in HRQoL, incidence of microvascular complications, and control of haemoglobin A_1c_ (HbA_1c_) had positive standardized B‐W scores and mean B‐W scores (Table 3). Therefore, the information above was more frequently regarded as the most needed. Among the attributes, patients were most interested in the incidence of macrovascular complications. In contrast, information on pill burden, weight change, and dosing frequency appeared to be less important. Results of the sensitivity analysis showed that the ranking probability of attributes was similar between the mean B‐W score and the SUCRA score (Supporting Information S1: Appendix 5). Attributes of information needs on comparative effectiveness had a higher ranking and SUCRA value than those in other domains (Figure 2).

**Table 3:** BW scaling estimates by count‐based analysis.

Attributes	Best	Worst	B‐W score	Standardized B‐W score	Mean B‐W score		SUCRA score	
					Mean	SE	Mean	SE
Incidence of macrovascular complications	658	25	633	0.130	1.174	0.040	0.933	0.031
Length of extended life years	583	57	526	0.108	0.976	0.047	0.857	0.020
Changes in HRQoL	513	66	447	0.092	0.829	0.043	0.791	0.012
Incidence of microvascular complications	361	38	323	0.075	0.599	0.037	0.689	0.010
Control of HbA_1c_	403	85	318	0.066	0.590	0.046	0.686	0.010
Incidence of severe hypoglycaemia events	143	204	−61	−0.014	−0.113	0.040	0.424	0.005
Out‐of‐pocket costs	322	395	−73	−0.015	−0.135	0.059	0.418	0.012
Incidence of gastrointestinal side events	99	205	−106	−0.022	−0.197	0.035	0.389	0.012
Dosing frequency	62	691	−629	−0.130	−1.167	0.040	0.131	0.003
Weight change	53	737	−684	−0.141	−1.269	0.041	0.096	0.010
Pill burden	37	731	−694	−0.161	−1.288	0.035	0.088	0.007

Abbreviations: B‐W, best‐minus‐worst; HbA_1c_, haemoglobin A_1c_; HRQoL, health‐related quality of life; SE, standard error; SUCRA, surface under the cumulative ranking.

### 3.3 BWS estimates by modelling approaches

Results of modelling approaches showed that the most desired information among diabetic patients remained the incidence of macrovascular complications, followed by length of extended life years, changes in HRQoL, and incidence of microvascular complications (Table 4). The ranking of attributes was identical between the two types of regression models. The findings from modelling approaches were consistent with the count‐based analysis.

**Table 4:** Best–worst scaling estimates by modelling approaches.

Attributes	Conditional logit model		Multinomial logit model	
	Coefficients	SE	Coefficients	SE
Incidence of macrovascular complications	3.070***	0.072	3.071***	0.071
Length of extended life years	2.672***	0.083	2.853***	0.072
Changes in HRQoL	2.553***	0.074	2.692***	0.071
Incidence of microvascular complications	2.514***	0.074	2.486***	0.073
Control of HbA_1c_	2.438***	0.072	2.420***	0.071
Incidence of severe hypoglycaemia events	1.547***	0.071	1.503***	0.069
Out‐of‐pocket costs	1.373***	0.066	1.459***	0.066
Incidence of gastrointestinal side events	1.350***	0.066	1.340***	0.066
Dosing frequency	0.282***	0.059	0.286***	0.060
Weight change	0.263***	0.059	0.245***	0.059
Pill burden	Ref		Ref	

Abbreviations: HbA_1c_, haemoglobin A1c; HRQoL, health‐related quality of life; Ref, reference; SE, standard error.

*** *p* < .001.

### 3.4 Heterogeneity of patient information needs

The distribution of the B‐W score didn't satisfy the normal distribution, indicating heterogeneity in patient information needs (Supporting Information S1: Appendix 6). Therefore, the subgroup analysis of preference heterogeneity was warranted. Our results showed that the top five aspects of information that patients valued belonged to the domain of comparative effectiveness (Supporting Information S1: Appendices 7 and 8). The incidence of macrovascular complications was invariably the primary concern.

Patients who were less than 65 years old ranked the length of extended life years as the second priority of their information needs. Nevertheless, older patients had a stronger desire to know the incidence of microvascular complications. Aside from the incidence of macrovascular complications and length of extended life years, patients with satisfied glycemic control were more eager to obtain information on changes in HRQoL. Patients with poor glycemic control paid more attention to the incidence of microvascular complications. As far as diabetes complications are concerned, patients without complications were more interested in whether the new antidiabetic medications could prolong life. However, patients with diabetes complications had an urge to identify the incidence of microvascular complications. In addition to the information on comparative effectiveness, patients with poor glycemic control or diabetes complications cared more about the incidence of severe hypoglycaemia events. They had a greater interest in recognizing the incidence of gastrointestinal side events as well.

## 4 DISCUSSION

SDM is defined as an approach where clinicians and patients make decisions together using the best available evidence.^38^ An essential requirement for the implementation of a successful SDM process is full awareness of patient information needs.^39^ However, patient information needs for new antidiabetic medications have seldom been reported. BWS offers a straightforward and transparent, patient‐centred approach to assess the relative importance of attributes. The methodology extends insights to information needs and can directly support SDM by indicating what should be considered a priority in a list of information. As far as we know, this is the first BWS experiment to investigate patient information needs for new antidiabetic medications. Furthermore, we provide an in‐depth exploration of the information preference heterogeneity.

To assess patient information needs for new antidiabetic medications, we classified 11 attributes to reflect information needs into four domains. Our estimation revealed a clear dominance of the comparative effectiveness domain. The domain had five attributes, including the incidence of macrovascular events, length of extended life years, changes in HRQoL, incidence of microvascular complications, and control of HbA_1c_. The relative importance of specific information needs on two domains, comparative safety (i.e., the incidence of severe hypoglycaemia events, the incidence of gastrointestinal side events) and affordability (i.e., out‐of‐pocket costs), was secondary to the comparative effectiveness. Information needs on convenience (i.e., dosing frequency, pill burden) and weight change were the least important.

Patient information needs on the comparative effectiveness of new medications were consistent with prior studies on patient preferences for antidiabetic therapeutics. For example, a survey showed that cardiovascular health and clinical benefits were most valued among diabetic patients when selecting second‐line antidiabetic medications.^40^ Treatment outcomes, including the control of HbA_1c_, glycemic stability, and cardiovascular events, were highly valued by diabetic patients in another survey.^41^

A new finding in our study is that to facilitate the SDM, patients had the greatest interest in whether new antidiabetic medications could reduce the incidence of macrovascular complications. Diabetes causes a variety of macrovascular complications, such as coronary heart disease, arrhythmias and sudden death, cerebrovascular disease, and peripheral artery disease.^42^ Macrovascular complications are the leading cause of mortality and decreased quality of life.^30^ Macrovascular complications and mortality are considered important clinical trial endpoints for evaluating the efficacy of antidiabetic medications.^43^ Another new finding in our study is that patients expected to be informed about the changes in HRQoL. Diabetes considerably affects all dimensions of patients' daily lives and leaves them vulnerable to deteriorated HRQoL. A systematic review showed that HRQoL had been viewed as an essential attribute in value‐assessment frameworks for new medications.^44^ Therefore, information on changes in HRQoL is essential for patients making decisions about antidiabetic therapies.

Patient populations are heterogeneous, and one patient's information needs may differ from those of another.^45^ We performed a subgroup analysis and noticed that patients who were older, had poor glycemic control, or had diabetes complications paid more attention to the incidence of microvascular complications. Moreover, patients with poor glycemic control or with diabetes complications care more about the incidence of severe hypoglycaemia events and the incidence of gastrointestinal side events. Potential reasons for preference heterogeneity are that patients with a poor prognosis are more likely to be exposed to a high risk of complications and adverse events.^46^, ^47^, ^48^, ^49^ Hence, the progression of disease has a significant bearing on patient information needs.

Currently, there remain difficulties for patients to be involved in SDM, such as inadequate knowledge, support, and resources in the decision‐making process.^50^ Results from our survey revealed very poor patients’ knowledge of their antidiabetic medications, with only a few patients claiming they had full awareness of the medications. Nevertheless, the majority of respondents in our study declared their desire to participate in SDM and expressed their expectation of the use of PDAs to know the value of new antidiabetic medications. Previous studies showed that PDAs can make people more knowledgeable and better informed when facing health treatment, thus having a more active role in decision‐making.^51^, ^52^ The patients, once fully informed, would be able to participate in clinical decisions to the extent that they desired.

Information on comparative effectiveness is critical for diabetic patients. To promote SDM, evidence on the incidence of macrovascular complications, length of extended life years, changes in HRQoL, and incidence of microvascular complications should be provided to patients. PDAs might visually outline validated and reliable evidence to indicate potential values like comparative effectiveness, comparative safety, affordability, and the convenience of new antidiabetic medications. Given the variations in patient information needs, new personalized PDAs that consider patients' priorities in the information needs of new antidiabetic medications are suggested to be developed in the future.

Several limitations of our study should be acknowledged. First, we used a series of domains and attributes that were identified from the literature review and focus group discussion. Owing to the methodological requirements of BWS, our analysis was unable to involve other attributes that might also be meaningful. Second, the Case 1 BWS is a parsimonious method, and the trade‐offs between various levels of attributes remain unobserved. Third, there is currently no consensus regarding the optimal sample size for the BWS experiment. Although the sample size in our study is adequate according to the rule of thumb, we remain unclear about the influence of the sample size on our major findings. Fourth, although we tried our best to minimize the bias caused by technical language and phrasing during the survey, there were still a few patients excluded due to a lack of confidence in making their choices. We used colourful pictures to interpret the choice scenarios and compiled survey manuals for further explanation. More effective forms of questionnaires should be developed to ensure all patients can understand the choice scenarios that require high literacy. Finally, patients were recruited from Jiangsu Province, which might limit the generalizability. Future studies should have a nationally representative sample by including other regions in China.

Despite these limitations, our findings provide valid evidence of the information needs for new antidiabetic medications from the patient perspective. A patient‐centred approach for the management of diabetes has been increasingly recommended in China, and fulfilling patient information needs is a fundamental aspect of patient‐centred care. The major contributions of our study are as follows. First, our study would not only inform patient‐clinician SDM but also be helpful for guiding the development of PDAs that can better satisfy patient information needs. Second, we performed a one‐to‐one, face‐to‐face BWS survey that followed good research practices and offered the advantage of measuring patient information needs concerning the value of new antidiabetic medications. In addition to generating implicit rank information, BWS can produce norms with higher predictive validity than other response formats and require less data to be collected.^53^ Third, we captured the heterogeneity of information needs, which can be used to add patient‐centred evidence to improve the quality of diabetic care. Finally, our study highlights the significance of meeting patient information needs when implementing SDM, therefore increasing patient involvement in decisions and better aligning antidiabetic therapies with patients' individual priorities.

## 5 CONCLUSIONS

In summary, our study suggests that comparative effectiveness is the most desirable information domain among diabetic patients. Patients are most concerned about whether the new antidiabetic medications can reduce the incidence of macrovascular complications. The relative importance of information regarding specific comparative safety and affordability domains was secondary to comparative effectiveness. Patient information needs vary by age, glycemic control status, and whether they have diabetes complications. Clinicians should be fully aware of patient information needs on the value of new antidiabetic medications, provide evidence as required by patients, and make patient‐centred decisions. PDAs that integrate scientific evidence to reflect the multi‐attribute value of new antidiabetic medications would be helpful to support the SDM process.

## AUTHOR CONTRIBUTIONS

**Tongling Xie**: Conceptualization; investigation; methodology; formal analysis; writing—original draft; resources. **Jingyi Meng**: Data curation; investigation; formal analysis; software. **Zhe Feng**: Methodology; visualization. **Yue Gao**: Investigation. **Tian Chen**: Investigation. **Yalan Chen**: Project administration; writing—original draft. **Jinsong Geng**: Conceptualization; funding acquisition; methodology; formal analysis; writing—review and editing; validation; supervision. All authors read and approved the final version of the manuscript.

## CONFLICT OF INTEREST STATEMENT

The authors declare no conflict of interest.

## ETHICS STATEMENT

This study, including the patient consent process, has been approved by the Medical Ethics Committee at Nantong University (Ethical Approval‐2021069) and conforms to the ethical guidelines of the Declaration of Helsinki. Informed, written consent was obtained from all patients before their participation in the study.

## Supporting information

Supporting information.

## Data Availability

The data that support the findings of this study are available from the corresponding author upon reasonable request.
