# Supplementary material for: Assessing patient information needs for new antidiabetic medications to inform shared decision‐making: A best–worst scaling experiment in China
Source: Health Expect. 2024 Apr 30;27(3):e14059. doi: 10.1111/hex.14059 (PMC11061543; doi:10.1111/hex.14059)
Supplement: Supplementary file 1 — Supporting information. [file HEX-27-e14059-s001.pdf]

Appendix S1: Literature review of value assessment frameworks for new health technologies

Table S1 Domains of the value assessment frameworks for new health technologies

ID	Perspective	Clinical Benefits	PROs	Safety/ Tolerability	Economics/ Affordability	Convenience
Angelis A 2016 [1]	Society	√	√	√	√	√
Angelis A 2017 [2]	Society	√	√	√	√	√
Gilabert-Perramon A 2017 [3]	Patients	√	√	√	√	—
Kristensen FB 2017 [4]	Society	√	—	√	√	—
Lakdawalla DN 2018 [5]	Society	√	√	—	√	√
Roldán ÚB 2018 [6]	Pharmacy and therapeutics committee	√	√	√	√	√
Wagner M 2018 [7]	Patients and clinicians	√	√	√	√	√
Hsu JC 2019 [8]	Society	√	√	√	√	√
Camps C 2020 [9]	Oncologists	√	√	√	√	—
Ezeife DA 2020 [10]	Society	√	√	√	√	—
Vandewalle B 2021 [11]	Society	√	√	√	√	—

Note: "√": the domain was involved in the value assessment framework; "—": the domain was not mentioned in the framework; PROs, Patient-reported Outcomes

Appendix S2: Literature review of attributes regarding anti-diabetic medications in preference-based studies

Table S2 Attributes of anti-diabetic medications in preference-based studies

Attributes	References
Incidence of macrovascular events	12-17
Life expectancy	18,19
Incidence of microvascular events	18
Control of HbA_{1c}	12-15,18,20-26
Incidence of hypoglycemic events	13-16,19,21-25,27
Out-of-pocket cost	13,15,18,22,23,28
Incidence of gastrointestinal side effects	15,20-22,27,29
Dosing Frequency	12,15,22,26,27
Weight change	12-15,17,19,20,22,23,27,28,30
Dosage	22,28
Mode of administration	13,15,20,22,29

Abbreviations: HbA_{1c}, glycated hemoglobin

References

1. Angelis A, Kanavos P. Value-based assessment of new medical technologies: towards a robust methodological framework for the application of multiple criteria decision analysis in the context of health technology assessment. *PharmacoEconomics.* 2016;34(5):435-446. doi: 10.1007/s40273-015-0370-z
2. Angelis A, Kanavos P. Multiple criteria decision analysis (MCDA) for evaluating new medicines in health technology assessment and beyond: the Advance Value Framework. *Soc Sci Med.* 2017;188:137-156. doi: 10.1016/j.socscimed.2017.06.024
3. Gilabert-Perramon A, Torrent-Farnell J, Catalan A, et al. Drug evaluation and decision making in catalonia: Development and validation of a methodological framework based on multi-criteria decision analysis (MCDA) for orphan drugs. *Int J Technol Assess Health Care.* 2017;33(1):111-120. doi: 10.1017/S0266462317000149
4. Kristensen FB, Lampe K, Wild C, Cerbo M, Goettsch W, Becla L. The HTA Core Model®- 10 years of developing an international framework to share multidimensional value assessment. *Value Health.* 2017;20(2):244-250. doi: 10.1016/j.jval.2016.12.010
5. Lakdawalla DN, Doshi JA, Garrison LP, Phelps CE, Basu A, Danzon PM. Defining elements of value in health care-a health economics approach: an ISPOR Special Task Force report [3]. *Value Health.* 2018;21(2):131-139. doi: 10.1016/j.jval.2017.12.007
6. Roldán Ú B, Badia X, Marcos-Rodríguez JA, et al. Multi-criteria decision analysis as a decision-support tool for drug evaluation: a pilot study in a pharmacy and therapeutics committee setting. *Int J Technol Assess Health Care.* 2018;34(5):519-526. doi: 10.1017/S0266462318000569
7. Wagner M, Samaha D, Cuervo J, et al. Applying reflective multicriteria decision analysis (MCDA) to patient-clinician shared decision-making on the management of gastroenteropancreatic neuroendocrine tumors (GEP-NET) in the Spanish context. *Adv Ther.* 2018;35(8):1215-1231. doi: 10.1007/s12325-018-0745-6
8. Hsu JC, Lin JY, Lin PC, Lee YC. Comprehensive value assessment of drugs using a multi-criteria decision analysis: An example of targeted therapies for metastatic colorectal cancer treatment. *PLoS ONE.* 2019;14(12):e0225938. doi: 10.1371/journal.pone.0225938
9. Camps C, Badia X, García-Campelo R, et al. Development of a multicriteria decision analysis framework for evaluating and positioning oncologic treatments in clinical practice. *JCO Oncol Pract.* 2020;16(3):e298-e305. doi: 10.1200/JOP.19.00487
10. Ezeife DA, Dionne F, Fares AF, et al. Value assessment of oncology drugs using a weighted

criterion-based approach. *Cancer.* 2020;126(7):1530-1540. doi: 10.1002/cncr.32639

11. Vandewalle B, Amorim M, Ramos D, et al. Value-based decision-making for orphan drugs with multiple criteria decision analysis: burosumab for the treatment of X-linked hypophosphatemia. *Curr Med Res Opin.* 2021;37(6):1021-1030. doi: 10.1080/03007995.2021.1904861
12. Mansfield C, Sikirica MV, Pugh A, et al. Patient preferences for attributes of type 2 diabetes mellitus medications in Germany and Spain: an online discrete-choice experiment survey. *Diabetes Ther.* 2017;8(6):1365-1378. doi: 10.1007/s13300-017-0326-8
13. Morillas C, Feliciano R, Catalina PF, et al. Patients' and physicians' preferences for type 2 diabetes mellitus treatments in Spain and Portugal: a discrete choice experiment. *Patient Prefer Adherence.* 2015;9:1443-1458. doi: 10.2147/PPA.S88022
14. Crossnohere NL, Janse S, Janssen E, Bridges JFP. Comparing the preferences of patients and the general public for treatment outcomes in type 2 diabetes mellitus. *Patient.* 2021;14(1):89-100. doi: 10.1007/s40271-020-00450-7
15. Liu S, Liu J, Yu Y, et al. What is valued most by patients with type 2 diabetes mellitus when selecting second-line antihyperglycemic medications in China. *Front Pharmacol.* 2021;12:802897. doi: 10.3389/fphar.2021.802897
16. Mühlbacher AC, Sadler A, Juhnke C. Personalized diabetes management: what do patients with diabetes mellitus prefer? A discrete choice experiment. *Eur J Health Econ.* 2021;22(3):425-443. doi: 10.1007/s10198-021-01264-6
17. Brooks A, Langer J, Tervonen T, Hemmingsen MP, Eguchi K, Bacci ED. Patient preferences for GLP-1 receptor agonist treatment of type 2 diabetes mellitus in Japan: a discrete choice experiment. *Diabetes Ther.* 2019;10(2):735-749. doi: 10.1007/s13300-019-0591-9
18. Donnan JR, Johnston K, Chibrikov E, et al. Capturing adult patient preferences toward benefits and risks of second-line antihyperglycemic medications used in type 2 diabetes: a discrete choice experiment. *Can J Diabetes.* 2020;44(1):6-13. doi: 10.1016/j.jcjd.2019.04.014
19. Mühlbacher A, Bethge S. What matters in type 2 diabetes mellitus oral treatment? A discrete choice experiment to evaluate patient preferences. *Eur J Health Econ.* 2016;17(9):1125-1140. doi: 10.1007/s10198-015-0750-5
20. Igarashi A, Bekker Hansen B, Langer J, et al. Preference for oral and injectable GLP-1 RA therapy profiles in Japanese patients with type 2 diabetes: a discrete choice experiment. *Adv Ther.* 2021;38(1):721-738. doi: 10.1007/s12325-020-01561-1
21. Janssen EM, Hauber AB, Bridges JFP. Conducting a discrete-choice experiment study following recommendations for good research practices: an application for eliciting patient preferences for

diabetes treatments. *Value Health.* 2018;21(1):59-68. doi: 10.1016/j.jval.2017.07.001

22. Janssen EM, Segal JB, Bridges JF. A framework for instrument development of a choice experiment: an application to type 2 diabetes. *Patient.* 2016;9(5):465-479. doi: 10.1007/s40271-016-0170-3

23. Feher MD, Brazier J, Schaper N, Vega-Hernandez G, Nikolajsen A, Bøgelund M. Patients' with type 2 diabetes willingness to pay for insulin therapy and clinical outcomes. *BMJ Open Diabetes Res Care.* 2016;4(1):e000192. doi: 10.1136/bmjdrc-2016-000192

24. Mol PG, Arnardottir AH, Straus SM, et al. Understanding drug preferences, different perspectives. *Br J Clin Pharmacol.* 2015;79(6):978-987. doi: 10.1111/bcp.12566

25. Gelhorn HL, Bacci ED, Poon JL, Boye KS, Suzuki S, Babineaux SM. Evaluating preferences for profiles of glucagon-like peptide-1 receptor agonists among injection-naive type 2 diabetes patients in Japan. *Patient Prefer Adherence.* 2016;10:1337-1348. doi: 10.2147/PPA.S109289

26. Poulos C, González JM, Lee LJ, et al. Physician preferences for extra-glycemic effects of type 2 diabetes treatments. *Diabetes Ther.* 2013;4(2):443-459. doi: 10.1007/s13300-013-0046-7

27. Fifer S, Rose J, Hamrosi KK, Swain D. Valuing injection frequency and other attributes of type 2 diabetes treatments in Australia: a discrete choice experiment. *BMC Health Serv Res.* 2018;18(1):675. doi: 10.1186/s12913-018-3484-0

28. Hauber AB, Tunceli K, Yang JC, et al. A survey of patient preferences for oral antihyperglycemic therapy in patients with type 2 diabetes mellitus. *Diabetes Ther.* 2015;6(1):75-84. doi: 10.1007/s13300-015-0094-2

29. Marchesini G, Pasqualetti P, Anichini R, et al. Patient preferences for treatment in type 2 diabetes: the Italian discrete-choice experiment analysis. *Acta Diabetol.* 2019;56(3):289-299. doi: 10.1007/s00592-018-1236-6

30. Andreadis P, Karagiannis T, Malandris K, et al. Semaglutide for type 2 diabetes mellitus: a systematic review and meta-analysis. *Diabetes Obes Metab.* 2018;20(9):2255-2263. doi: 10.1111/dom.13361

Appendix S3: Question guide for the focus group discussion

Focus Group Discussion

Objective

To identify attributes that could be used to investigate patient information needs for new anti-diabetic medications. The information attributes should reflect the multi-attribute value of new anti-diabetic medications. Our findings would inform clinicians to provide patients with the desired information, thus promoting shared decision-making.

Questions

1. Based on your personal clinical experience, what do diabetes patients usually want to know about their medications when you are making shared decisions with them?
2. According to the domains of the value assessment frameworks for new health technologies summarized from published literature, which domains do you think are essential for shared decision-making on anti-diabetic therapy?
3. According to the attributes regarding anti-diabetic medications in preference-based studies, which attributes do you think are necessary for shared decision-making on anti-diabetic therapy?
4. Based on the existing domains and attributes, what other attributes do you think need to be added?
5. Please provide some comments on the final attributes to be used in our study.

Appendix S4: Questionnaire for the best-worst scaling experiment (Translated version)

Survey site: ____________ Date: _____________ (Year/Month/Day)

Signature of the interviewer: ____________ Block: 1 Patient ID: ___________

Informed consent form

The purpose of this survey was to understand your information needs for new anti-diabetic medications. Your answers will be useful for improving diabetes clinical practice. Your personal information will be kept completely confidential.

Please fill out the survey informed consent form to indicate your agreement to participate.

Thank you for your cooperation and support!

Signature of the patient:

Assessing patient information needs for new anti-diabetic medications

Thank you for taking the time to fill out the questionnaire. The results of this questionnaire are only used for academic research. You do not need to worry about your privacy. Please answer according to your own feelings and perceptions. After completing the questionnaire, you will be rewarded with a gift.

If you do not answer carefully, you will provide incorrect information for healthcare decision-making, thus affecting your own interests.

Please tick "√" in "○" or "☐" when appropriate, and fill in "_______".

Thank you again for your kind support!

Part A: Personal information (For items 1 to 8, please refer to the electronic medical record)

1. Gender: ○ Female ○ Male

2. Age: _______Years old

3. Type of patient: ○ Inpatient ○ Outpatient

4. Years since the diagnosis of type 2 diabetes: ________ years _______ months (If just diagnosed, write "first time." If diagnosed within 1 year, the time is accurate to "month." If diagnosed more than one year, the time is accurate to "year.")

5. Does the patient have any of the following diabetes complications (heart, brain, kidney, peripheral nerves, eyes, feet, etc.)?

   ○ Yes ○ No

   If "Yes", please choose the complications (multiple choices):

   ☐ Cardiovascular complications ☐ Cerebrovascular Complications

   ☐ Diabetic foot ☐ Diabetic retinopathy ☐ Diabetic nephropathy

   ☐ Diabetic neuropathy ☐Other types of complications__________________________

6. Does the patient have other chronic diseases (e.g., high blood pressure, chronic respiratory disease, etc.)?

   ○ Yes ○ No

   If yes, please fill in the name of the disease: ______________________________

7. At present, the administration route of anti-diabetic drugs for the patient:

☐ Oral ☐ Injection ☐ Other routes: ____________________

The current frequency of drug administration: ____________________

8. The current glycemic control for the patient

○ Good ○ Fair ○ Poor

9. Level of education

○Unschooled ○Primary School ○Junior high school ○High school

○Junior college or Higher vocational college ○Bachelor's degree

○Master's degree or above

10. Occupation

○Civil Servant ○Staffing of public institution ○Company employee ○Factory worker

○Farmer ○Unemployed ○Retiree

○Freelancers (self-employed, temporary stallholder, freelance writer, etc.)

○Other type: _______________

11. Marital status

○Single ○Married ○Divorced ○Widowed

12. Monthly household income (gross income):

○≤2000 CNY ○2001~4000 CNY ○4001~6000 CNY ○6001~8000 CNY

○8001~10000 CNY ○10001~12000 CNY ○>12000 CNY

13. Type of health insurance:

☐Urban Employees Basic Medical Insurance

☐Urban-Rural Residents Basic Medical Insurance (including former New Rural Cooperative Medical Insurance, and former Urban Residents Basic Medical Insurance)

☐Commercial Health Insurance ☐Other types: ________________________

14. The overall satisfaction with your public health insurance:

○Very dissatisfied ○Dissatisfied ○Neither satisfied nor dissatisfied

○Fairly satisfied ○Very satisfied

15. I am fully aware of my anti-diabetic medications.

○Strongly disagree ○Disagree ○Neither agree nor disagree

○Agree ○Strongly agree

16. I am willing to actively engage in the shared decision-making process for anti-diabetic medications.

○Strongly disagree ○Disagree ○Neither agree nor disagree

○Agree ○Strongly agree

17. I am willing to know the value of new anti-diabetic medications with the help of patient decision aids, which provide scientific evidence.

○Strongly disagree ○Disagree ○Neither agree nor disagree

○Agree ○Strongly agree

Part B: Patient preferences for information needs on new anti-diabetic medications for inform shared decision-making

Assuming your clinical symptoms have not been well improved, you need to use new anti-diabetic medications. However, you are unclear about the value of the new anti-diabetic medications. Therefore, you need information on the new medications to actively participate in medical decisions with clinicians.

Please read all the items carefully and tick the boxes for the most needed information (most important) and the one that was the least needed (least important) for you.

Scenario 1

Most needed	Information		Least Needed
	**Incidence of macrovascular complications**	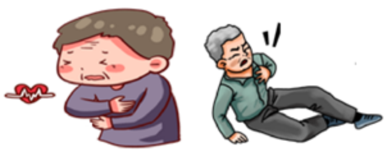<br>e.g., myocardial infarction, stroke	
	**Incidence of microvascular complications**	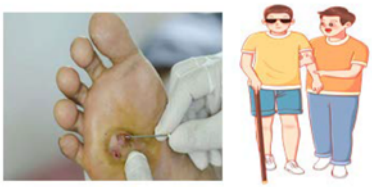<br>e.g., foot ulcer, blind	
	**Incidence of severe hypoglycemic events**	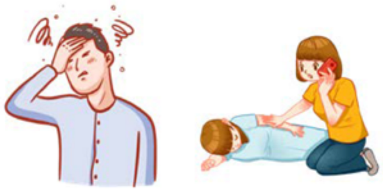<br>e.g., fatigue, dizziness, coma	
	**Incidence of gastrointestinal events**	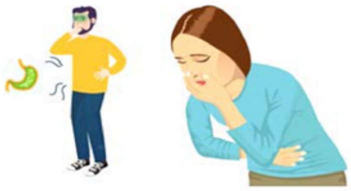<br>e.g., nausea, vomiting	

Scenario 2

Most needed	Information		Least Needed
	**Weight change**	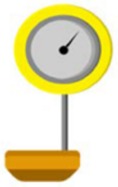 e.g., weight gain, weight loss	
	**Dosing frequency**	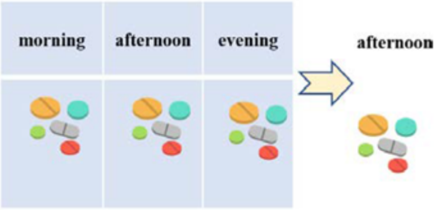	
	**Pill burden**	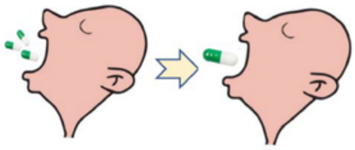	
	**Out-of-pocket costs**	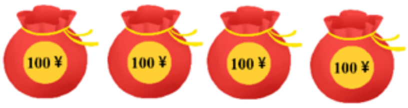	

Scenario 3

Most needed	Information		Least Needed
	**Control of HbA_{1c}**	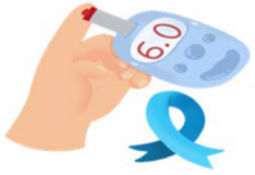	
	**Incidence of macrovascular complications**	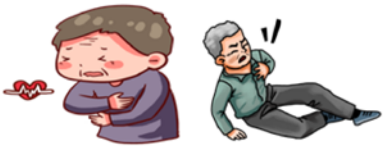 e.g., myocardial infarction, stroke	
	**Incidence of severe hypoglycemic events**	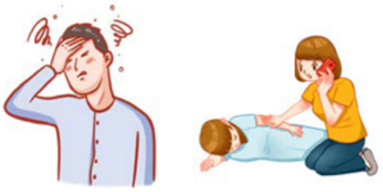 e.g., fatigue, dizziness, coma	
	**Length of extended life years**	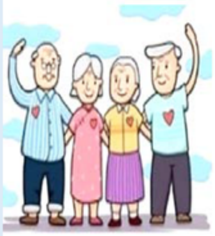 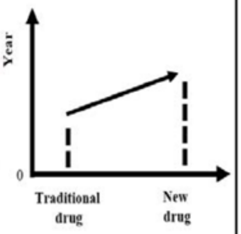	

Scenario 4

Most needed	Information	Least Needed
	**Control of HbA_{1c}** 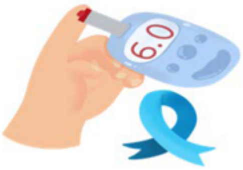	
	**Incidence of gastrointestinal events** 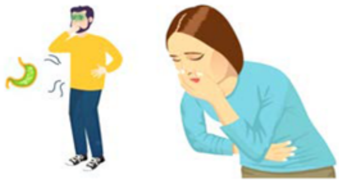 e.g., nausea, vomiting	
	**Pill burden** 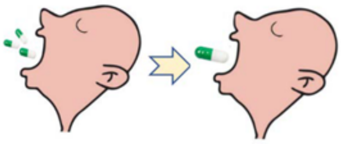	
	**Changes in health-related quality of life** 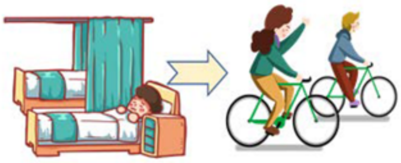 e.g., mobility, self-care, usual activities, pain/discomfort, anxiety/depression	

Scenario 5

Most needed	Information	Least Needed
	**Incidence of macrovascular complications**<br>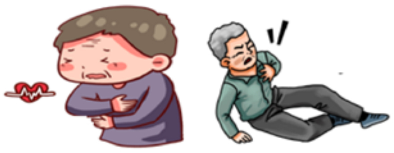<br>e.g., myocardial infarction, stroke	
	**Incidence of microvascular complications**<br>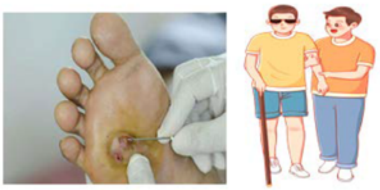<br>e.g., foot ulcer, blind	
	**Dosing frequency**<br>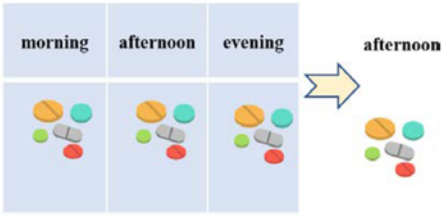	
	**Changes in health-related quality of life**<br>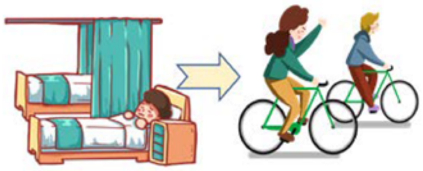<br>e.g., mobility, self-care, usual activities, pain/discomfort, anxiety/depression	

Scenario 6

Most needed	Information	Least Needed
	**Weight change** 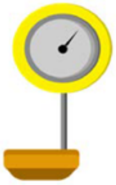 e.g., weight gain, weight loss	
	**Out-of-pocket costs** 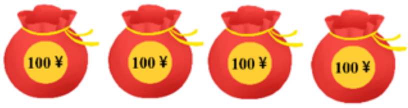	
	**Changes in health-related quality of life** 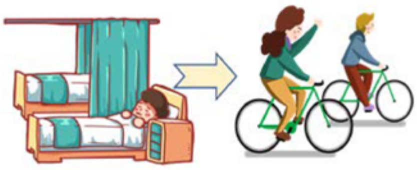 e.g., mobility, self-care, usual activities, pain/discomfort, anxiety/depression	
	**Length of extended life years** 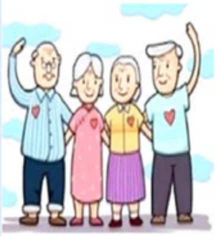 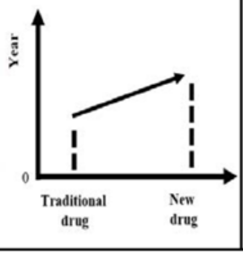	

Please select the score from zero to 10 and give a tick "√" in the score to reflect your confidence when making choices from Scenario 1 to Scenario 6:

0: no confidence at all										10: very confident
0	1	2	3	4	5	6	7	8	9	10

- 8 -

Appendix to the questionnaire: assessment of health-related quality of life

1. Please "√" in the following table according to your actual feeling and health condition

	Best →				Worst
	No problems	Slight problems	Moderate problems	Severe problems	Unable to do
①Mobility					
②Self-care (e.g. washing, dressing yourself)					
③Usual activities (e.g. work, study, housework, family or leisure activities)					

	Best →				Worst
	No	Slightly	Moderately	Severely	Extremely
④Pain/Discomfort					
⑤Anxiety/Depression					

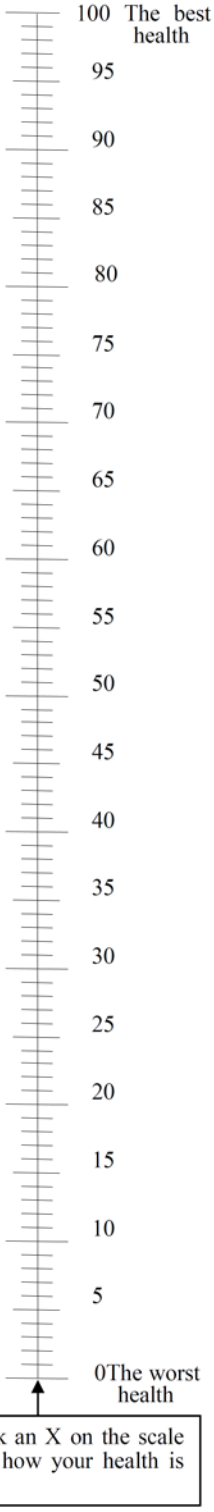

2. Mark an X on the scale to indicate how your health

- 100 means the best health you can imagine.
- 0 means the worst health you can imagine.

Please write the number you marked on the scale in the box below.

Your health today = ☐

Source of the measurement scale: EuroQol Research Foundation. EQ-5D-5L User Guide, 2019. Available from: https://euroqol.org/publications/user-guides

Appendix S5: Ranking probabilities and surface under the cumulative ranking plot

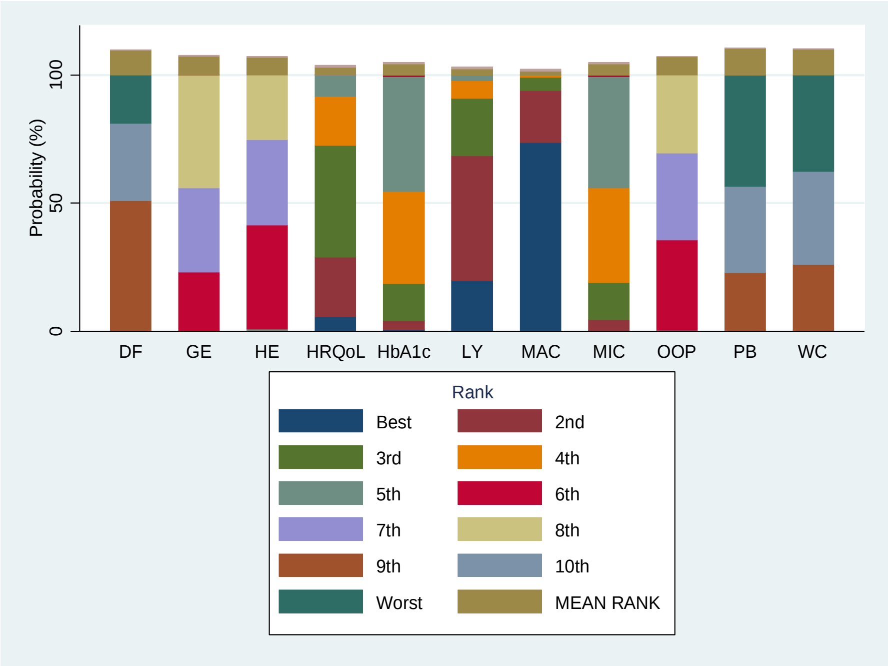

Figure S1 Ranking probabilities of each attribute

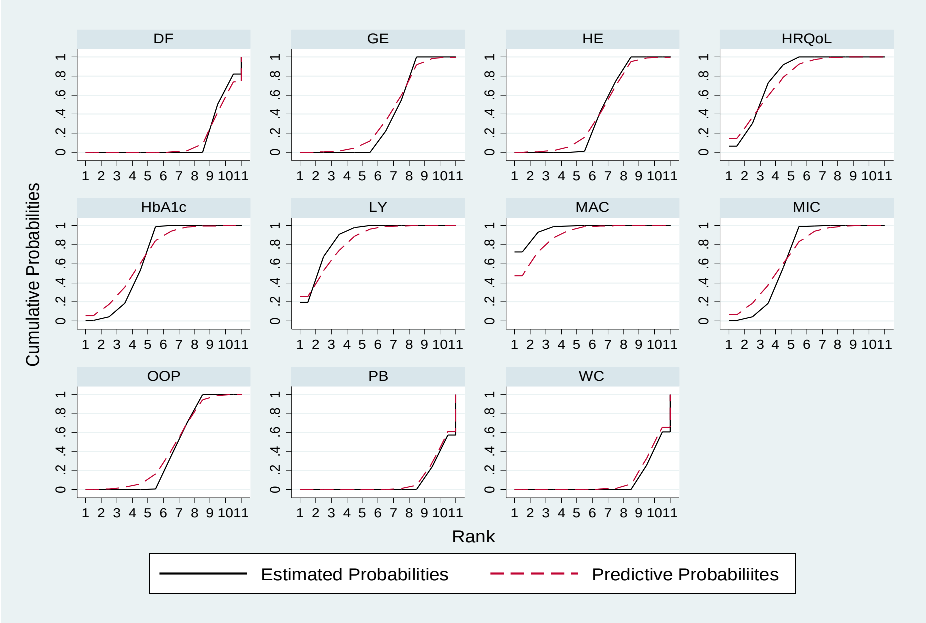

Figure S2 Surface under the cumulative ranking plot of estimated and predictive ranking probabilities

Abbreviations: DF, dosing frequency; GE, incidence of gastrointestinal side events; HE, incidence of severe hypoglycemic events; HRQoL, changes in health-related quality of life; HbA_{1c}, control of glycated hemoglobin; LY, length of extended life years; MAC, incidence of macrovascular complications; MIC, incidence of microvascular complications; OOP, out-of-pocket costs; PB, pill burden; WC, weight change

Appendix S6: Best-worst scaling score distribution of each attribute

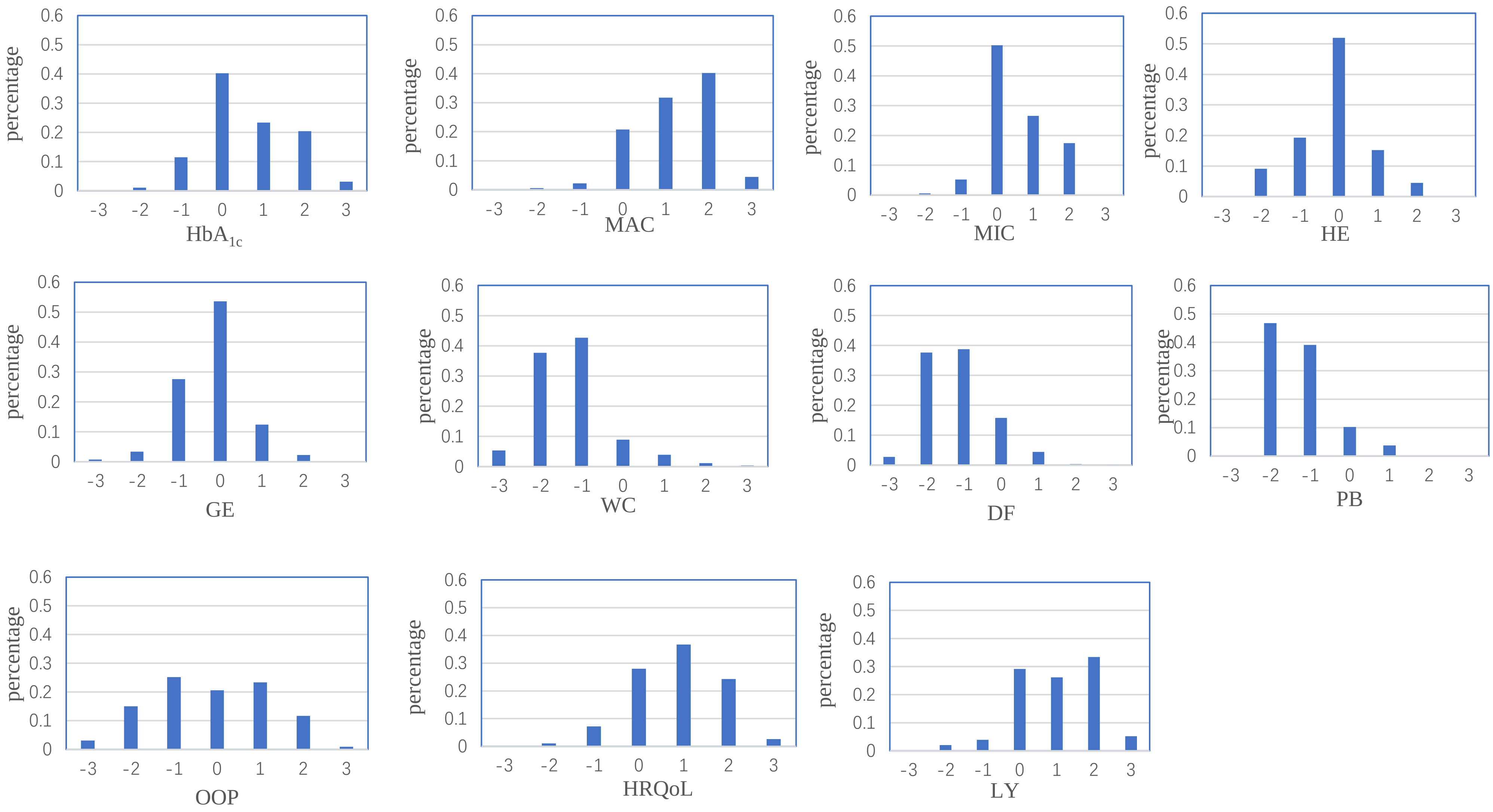

Figure S3 The best-worst scaling score distribution of each attribute

Abbreviations: HbA$_{1c}$, control of glycated hemoglobin; MAC, incidence of macrovascular complications; MIC, incidence of microvascular complications; HE, incidence of severe hypoglycemic events; GE, incidence of gastrointestinal side events; WC, weight change; DF, dosing frequency; PB, pill burden; OOP, out-of-pocket costs; HRQoL, changes in health-related quality of life; LY, length of extended life years

Appendix S7: Best-worst scaling estimates in subgroup analysis

Table S3 Best-worst scaling estimates in subgroup analysis by conditional logit regression

Attributes	Age		Glycemic control		Diabetes complications	
	＜65 years	≥65 years	Poor controlled	Well controlled	With complications	Without complications
Incidence of macrovascular complications	2.960***	3.296***	2.981***	3.214***	2.924***	3.352***
Length of extended life years	2.674***	2.684***	2.577***	2.847***	2.419***	3.145***
Changes in HRQoL	2.622***	2.450***	2.498***	2.661***	2.500***	2.694***
Incidence of microvascular complications	2.430***	2.734***	2.533***	2.509***	2.658***	2.324***
Control of HbA_{1c}	2.363***	2.613***	2.400***	2.511***	2.445***	2.479***
Incidence of severe hypoglycemic events	1.651***	1.366***	1.464***	1.690***	1.421***	1.778***
Out-of-pocket costs	1.361***	1.401***	1.170***	1.723***	1.080***	1.909***
Incidence of gastrointestinal side events	1.388***	1.284***	1.326***	1.397***	1.368***	1.354***
Dosing frequency	0.302***	0.251*	0.226**	0.375***	0.264***	0.312**
Weight change	0.398***	0.017	0.166*	0.422***	0.072	0.577***
Pill burden	Ref	Ref	Ref	Ref	Ref	Ref

Abbreviations: HRQoL, health-related quality of life; HbA_{1c}, glycated hemoglobin; Ref, reference; *p<0.05, **p<0.01, ***p<0.001

Appendix S8: Ranking of attributes in subgroup analysis

Table S4 Ranking of attributes in subgroup analysis by conditional logit regression

Attributes	Age		Glycemic control		Diabetes complications	
	<65 years	≥65 years	Poor controlled	Well controlled	With complications	Without complications
Incidence of macrovascular complications	1	1	1	1	1	1
Length of extended life years	2	3	2	2	5	2
Changes in HRQoL	3	5	4	3	3	3
Incidence of microvascular complications	4	2	3	5	2	5
Control of HbA_{1c}	5	4	5	4	4	4
Incidence of severe hypoglycemic events	6	7	6	7	6	7
Out-of-pocket costs	8	6	8	6	8	6
Incidence of gastrointestinal side events	7	8	7	8	7	8
Dosing frequency	10	9	9	10	9	10
Weight change	9	10	10	9	10	9
Pill burden	Ref	Ref	Ref	Ref	Ref	Ref

Abbreviations: HRQoL, health-related quality of life; HbA_{1c}, glycated hemoglobin; Ref, reference
